# Letter from the New Editor-in-Chief: My Perspective on *Environmental Health Perspectives*

**DOI:** 10.1289/ehp.1510617

**Published:** 2015-09-01

**Authors:** Sally Perreault Darney

**Affiliations:** Editor-in-Chief, *Environmental Health Perspectives*

As I come onboard as Editor-in-Chief of *EHP* this month, I’d like to share my thoughts with you about a vision for taking *EHP* to the next level. With an impact factor of 7.98, *EHP* has become the “go to” journal for research and news about each sector of environmental health. Now is the time to link these sectors and bring systems thinking to bear on the field.

Inter- and transdisciplinary research approaches are being applied to bridge the many factors in the built and natural environments that interact to impact health, including a complex array of social determinants. Real-world scenarios, where environmental risks and benefits are all too often unevenly distributed, also bring environmental justice to the research stage. Advances in our mechanistic understanding of how chemical, physical, and psychosocial stressors interact to influence our biological responses, for better or worse, provide avenues for more informed risk assessment, innovative intervention strategies, and more effective policy making. New approaches for monitoring and modeling environmental exposures are helping us better assess, predict, and prevent potentially dangerous exposures. Innovative personal monitors and web-based geospatial tools are putting data and information in the hands of scientists, regulators, community decision makers, and the public alike. What a great time to be in the environmental sciences! What a fabulous opportunity for *EHP* to be the platform that brings this research to our stakeholders!

The challenge, as I see it, is for *EHP* to make new knowledge accessible in forms that are “fit for purpose” for the scientific community, for regulatory application at national and local scales, and for the public to inform personal and community-based decisions. “Fit for purpose” means not only understandable by diverse audiences but also globally accessible. We can do this by marrying new data sharing and contemporary social networking tools in today’s digital age with traditional scholarly publishing.

This is a tall order for an Editor-in-Chief. Who am I to take on this important job? I bring to the *EHP* table more than 30 years of health effects research and organizational leadership experience in reproductive toxicology, environmental epidemiology, and children’s health, gained in the U.S. Environmental Protection Agency (EPA) Office of Research and Development and through service as president and board member of two scientific societies. I came to the EPA in 1984 with a PhD in Anatomy and Reproductive Biology from the University of Hawaii John A. Burns School of Medicine and postdoctoral training at the Johns Hopkins Bloomberg School of Public Health. As a bench scientist I published extensively under the name Sally D. Perreault.

After many years of conducting research (and managing staff), I stepped away from the bench to work first as a National Program Director and then as an Associate Director in the Immediate Office of the Office of Research and Development. In these roles I helped shape two new National Research Programs: *1*) Chemical Safety for Sustainability and *2*) Sustainable and Healthy Communities. Serving in these roles also expanded the scope of my experience across disciplines to include principles of exposure science, engineering solutions, computational and predictive toxicology, community-based participatory research, and environmental justice.

With respect to editorial experience, I have served as an Associate Editor for *Biology of Reproduction* and Coeditor-in-Chief of the *Journal of Andrology* during critical times in these journals’ histories, as they transitioned to electronic publication. So I understand the importance of critical peer review and publication ethics in ensuring scientific rigor, as well as efficiency (short turn-around times) to meet author requirements for career development. Above all, I believe in treating each submission with respect, reflecting the professional efforts of each author.

Thankfully, I will not be going it alone in this new role. My predecessor, Hugh Tilson, deserves special recognition for bringing *EHP* to new heights as Editor-in-Chief from 2008 through 2014. Our new Operations Manager, Shaun Halloran, brings invaluable experience in scholarly publication based on many years of experience managing several families of scientific journals, so we can expect to realize big gains in publishing practices and efficiency. And we are all grateful to Jane Schroeder, who generously served as Interim Editor-in-Chief this past year. Jane’s expertise in epidemiology will complement mine in toxicology as we move forward. With the entire *EHP* staff, I will explore new ways to improve journal management efficiency with modern methods and tools.

Finally, I want to acknowledge the members of the current *EHP* Advisory Committee for their sage advice and, of course, thank our Associate Editors, whose commitment to fair and helpful peer review is so essential in publishing the best papers. I am forming a new Advisory Committee, and with their help I will update the membership of *EHP* ’s Board of Associate Editors and Editorial Review Board over the next month or two. Our goal with these updates is to increase the range of scientific capabilities across disciplines.

As I assume the new role of Editor-in-Chief, I welcome input from you, our readers and contributors. Together with the outstanding team of our boards and the *EHP* staff, I look forward to taking the journal to the next level.

